# Human Cytomegalovirus Antigens in Malignant Gliomas as Targets for Adoptive Cellular Therapy

**DOI:** 10.3389/fonc.2014.00338

**Published:** 2014-11-26

**Authors:** Daniel Landi, Meenakshi Hegde, Nabil Ahmed

**Affiliations:** ^1^Center for Cell and Gene Therapy, Baylor College of Medicine, Houston, TX, USA; ^2^Hematology and Oncology, Texas Children’s Cancer Center, Houston, TX, USA; ^3^Houston Methodist Hospital, Houston, TX, USA

**Keywords:** immunotherapy, cytomegalovirus, glioblastoma, adoptive cellular therapy

## Abstract

Malignant gliomas are the most common primary brain tumor in adults, with over 12,000 new cases diagnosed in the United States each year. Over the last decade, investigators have reliably identified human cytomegalovirus (HCMV) proteins, nucleic acids, and virions in most high-grade gliomas, including glioblastoma (GBM). This discovery is significant because HCMV gene products can be targeted by immune-based therapies. In this review, we describe the current level of understanding regarding the presence and role in pathogenesis of HCMV in GBM. We describe our success detecting and expanding HCMV-specific cytotoxic T lymphocytes to kill GBM cells and explain how these cells can be used as a platform for enhanced cellular therapies. We discuss alternative approaches that capitalize on HCMV infection to treat patients with HCMV-positive tumors. Adoptive cellular therapy for HCMV-positive GBM has been tried in a small number of patients with some benefit, but we reason why, to date, these approaches generally fail to generate long-term remission or cure. We conjecture how cellular therapy for GBM can be improved and describe the barriers that must be overcome to cure these patients.

## Background

Malignant gliomas are the most common primary brain tumor in adults, with over 12,000 new cases diagnosed in the United States each year (1). Glioblatoma (GBM) is the most common and lethal of the gliomas, and debilitating cognitive and motor deficits in patients who undergo surgical resection or radiation are common. Currently, the standard of care utilizing surgery, radiation, and the alkylating agent, temozolomide, leave GBM patients with a dismal median survival time of 15 months after diagnosis (1).

Although adoptive cellular therapies, particularly those using cytotoxic T cells (CTL), have been effective in treating disseminated viral infections (2) and Epstein-Barr Virus-associated malignancies (3, 4), successes in treating patients with solid tumors by targeting tumor-associated antigens (TAA) have been more tempered. A subversive and strongly suppressive tumor microenvironment coupled with a paucity of adequately presented tumor antigens has limited the *in vivo* impact in early efforts.

Over the last decade, investigators have reliably identified human cytomegalovirus (HCMV) proteins, nucleic acids, and virions in most high-grade gliomas. This discovery is significant because HCMV gene products can be targeted by immune-based therapies, offering a new therapeutic approach for patients with HCMV-positive GBM.

Human cytomegalovirus is a β-herpes virus tropic for human glial cells, and between 50 and 90% of the world’s population is infected (5, 6). In most people, the virus remains latent after a primary infection, quelled by an effective adaptive immune response. Virus-infected cells are the natural target of cytotoxic T lymphocytes, and while debate continues regarding the role HCMV might have in tumorigenesis or tumor progression in GBM, HCMV-encoded proteins are certainly among the most appealing TAA identified for GBM so far.

In this review, we describe the current level of understanding regarding the presence and role in pathogenesis of HCMV in GBM. We describe our success in detecting and expanding HCMV-specific CTLs to kill GBM cells. We discuss other important immune-based techniques for killing GBM and describe alternative approaches that capitalize on HCMV infection in a subset of GBM patients. Adoptive cellular therapy for HCMV-positive GBM has been tried in a handful of patients with some benefit, but we reason why, to date, these approaches generally fail to generate long-term remission or cure. We conjecture how cellular therapy for GBM can be improved and describe the barriers that must be overcome to cure these patients.

## HCMV Elements in Glioblastoma

Human cytomegalovirus DNA and proteins have been found in 90–100% of primary GBM samples, as well as medulloblastoma, colon, prostate, and breast cancers (6–10). While initial reports differed on the prevalence of HCMV early or late protein expression in GBM (11), or whether HCMV could be identified at all (12), more recent reports utilizing standardized detection methods suggest that most high-grade gliomas, particularly GBM, contain HCMV early and late proteins (7). The thickness of paraffin block sections is important for optimizing detection of HCMV proteins, and 6 μm is an accepted standard for this process (6).

Under optimal conditions for detecting low levels of expression, detection of HCMV proteins in adult GBM is usually reported in 80–100% of tumor samples (6, 8, 13–15). We found that a high proportion of GBMs in children also contain intermediate-early 1 (IE1) and pp65, although the rate of HCMV-protein expression in pediatric GBM was lower than reported values for adults (Corder, Ahmed et al., in review).

Although HCMV expression is ubiquitous in GBMs, virus-specific oligonucleotides are not observed in areas of necrosis or in healthy tissue outside the tumor margin (6–8, 11, 15). Using consensus methods to section and fix primary GBM samples (7), we have shown complete concordance of pp65 and IE-1 detection between immunohistochemistry and *in situ* hybridization techniques (15). IE1 has been found in over 90% of GBM samples, and immunoreactivity to IE1 is generally limited to the nuclei and perinuclear cytoplasm of GBM tumor cells (11). We have detected pp-65 primarily in a nuclear distribution in GBM cells, but pp65 does not appear to be as prevalent as IE1 in terms of detection within GBM samples and within individual tumor cells (15). Cobbs and colleagues have also consistently generated HCMV DNA and RNA in GBM samples (6).

Although astrocytic tumors appear to express HCMV most often, oligodendrocytic tumors and, to a lesser extent, ependymal tumors also express HCMV (8). Within GBM tumors, Sheurer and colleagues (8) found that 79% of tumor cells were positive for HCMV IE1 using an immunoreactive probe. Four percent of cells in non-tumor areas of tissue in patients with GBM were positive for IE1 using similar detection methods. While CMV nucleic acids and, likely, virions have been detected in patient samples (6), CMV gene expression in human and some animal models appears to be transient, and viral DNA is often not found in clonal cell lines derived from transformed foci (15, 16).

## Likely Role of HCMV in High-Grade Glioma Pathogenesis and Tumor Progression

Whether HCMV plays a role in the development of GBM from astrocytes is unknown. The prevalence of HCMV gene expression products in patient GBM samples and the apparent interplay between HCMV and GBM pathways suggest HCMV can promote tumor progression, if not help trigger GBM tumorigenesis. Reactivation of similar herpesviruses such as EBV and human herpesvirus-8 (HHV8) can lead to malignant transformation, and important cellular pathways in glioma biology are promoted by HCMV gene expression (6).

The CMV genome encodes roughly 200 proteins, many of which are not necessary for replication (17). HCMV gene transcription can be activated in myeloid cells (18) and astrocytic cells (6) in the setting of inflammation. Within GBM, HCMV-proteins appear to impact telomerase activity, cellular differentiation, apoptosis, and migration (19, 20). In particular, US28 is a HCMV-encoded G-protein coupled receptor found primarily in vascular endothelial cells of GBM tumors that generates immunomodulatory effects through increasing IL-6 production (21). US28 induces COX-2 expression and results in signal transducer and activation of transcription 3 (STAT3) phosphorylation. STAT3 activity increases production of vascular endothelial growth factor (VEGF) and IL-6, ultimately promoting tumor formation *in vivo* (22).

Interestingly, HCMV is particularly tropic to both monocytes, which give rise to central nervous system (CNS) macrophages and microgila, and CD133^+^ glioma stem cells (GSCs) (5). GSCs produce colony stimulating factor-1, transforming growth factor (TGF) β1, and macrophage inhibitory cytokine-1 – cytokines known to recruit macrophages/microglia (23). HCMV-expressing GSCs also produce HCMV IL-10, a viral homolog of human IL-10 (24). HCMV IL-10 – treated monocytes produce angiogenic VEGF and TGF-β (5). The overall effect is the induction of the M2 phenotype characteristic in GBM-associated macrophages and microglia. In this way, HCMV is implicated in promoting an M2 immunosuppressive phenotype in monocyte-derived cells in GBM.

The M2 phenotype is characterized by downregulation of major histocompatibility (MHC) machinery and co-stimulatory surface molecules, with simultaneous upregulation of immunoinhibitory molecules, specifically B7-H1 (25). In non-GBM patients, transcriptome analysis has shown that HCMV-infected monocytes display a unique M1/M2 polarization signature that was skewed toward the classical M1 activation phenotype (26). However, HCMV-induced M2-associated genes are also expressed in the presence of both NF-κB and phosphatidylinositol 3-kinase (27). Whether HCMV might exert an overall immune-promoting versus regulatory effect on the M1/M2 axis is unknown, but clearly the ultimate result in GBM patients is a powerful regulatory M2 phenotype.

In addition to stimulating VEGF and TGF-β, HCMV IL-10 also induces expression of the HCMV-protein IE1, a modulator of viral replication and transcription in monocytes (5). In monocytes harboring HCMV, IE1 expression was induced after exposure to HCMV IL-10, possibly potentiating a feed-forward mechanism whereby HCMV could produce an M1/M2 polarization signature that promotes viral dissemination and persistence (7). GBM cell lines exposed to persistent IE-1 expression also exhibited dysregulation of phosphatidylinositol 3-kinase/AKT activity, retinoblastoma protein (Rb) phosphorylation, and expression of the p53 family of proteins (28), but the precise onco-modulatory mechanisms of IE-1 and other HCMV proteins are incompletely understood.

Collectively, GBM cells are well-known to impair the immune response through inhibitory cytokines, including IL-10, prostaglandin-E2, and TGF-β (29). Inhibitory pathways involve STAT3 activity, and inhibition of phagocytosis and increased IL-10 secretion were reversed when the STAT3 pathway was blocked in GSCs (23).

In most healthy individuals, HCMV remains latent throughout the lifetime of the host. Bone marrow CD34^+^ progenitor cells have been identified as one site of HCMV latency and the latent viral genome is carried through the myeloid lineage as these cells differentiate (18). While the latent state was previously regarded as a quiescent state with little transcription, HCMV is now known to express numerous proteins in latent states as well (18, 30).

While HCMV proteins and nucleic acids appear in most GBMs, the precise nature of and effect of specific HCMV gene expression in these patients remain poorly understood. To date, active viral replication in human gliomas has not been detected. Overall, current consensus opinion is that HCMV appears to play a significant oncomodulatory role in GBM (7).

## Targeting CMV Antigens in Malignant Gliomas with Adoptive Cellular Therapy

Over the last 20 years, cellular therapies targeting GBM-associated antigens have shown increasing promise. Active immunization strategies have been predominant, and several groups have reported improved survival and occasional long-term remissions through these approaches (14). Interestingly, one reported patient who received a dendritic cell vaccine generated from autologous GBM tumor lysate had a robust CD8^+^ T-cell response to the HCMV epitope pp65 after one injection of the vaccine (31).

Active immunization requires an immunogenic TAA, and a sustained anti-tumor effect requires an element of continued stimulation *in vivo*. An active immunization approach that targets a ubiquitous viral epitope expressed in GBM cells could generate a more sustained, robust *in vivo* anti-tumor response. In this way, the presence of HCMV in GBM represents an important immunogenic target that can be used to improve cellular immunotherapy for GBM and other CMV-positive tumors.

Adoptive cellular therapy can generate a large number of GBM-specific effector cells, and these effector cells can be tested for specificity and cytotoxicity *ex vivo* prior to infusion into a patient with GBM. However, compared with active immunization, the number of studies using adoptive cellular therapy to target GBM is relatively small.

Our group generated polyclonal CMV-specific CTLs from CMV-seropositive GBM patients for adoptive immunotherapy. To evaluate whether IE1 and pp65 could be targeted by CMV-specific T cells in GBM patients, we first measured the frequency of pre-existing T cells targeting these antigens in a cohort of CMV-seropositive GBM patients. All 11 patients in our cohort had T cells specific for IE1 and pp65 assessed by IFN-γ enzyme-linked immunospot assay (ELISA), although the precursor frequency of pp65-specific CTLs was significantly lower than healthy donors. Immunohistochemistry analyses of the primary GBM samples from these 11 patients found IE1 in 10/11 (91%) and pp65 in 5/11 (45%) (15).

We successfully reactivated and expanded CMV-reactive clones using antigen-presenting cells (APS) transduced with an adenoviral vector encoding IE1 and pp65. Median T-cell expansion was 123-fold within 6 weeks of culture. We demonstrated the specific cytotoxic effect of patient-derived, CMV-specific T cell products against autologous GBM cells that were loaded with pp65 and IE1 using a standard chromium release assay (15).

Although we and others (15, 32, 33) have had excellent success expanding and reactivating autologous HCMV-specific T cells in CMV-seropositive patients, this is not possible for subjects who are HCMV-seronegative. Interestingly, a significant proportion of seronegative patients with GBM still have HCMV proteins in their tumors. Debate continues whether these HCMV gene products are significant in GBM patients who lack serologic evidence of HCMV infection based on conventional clinical assays (34). Recent work demonstrating CMV-specific memory T and B cells in seronegative patients indicates that traditional serology assays are incomplete for determining whether a patient has been infected with CMV (35).

In some instances, patients whose GBM contained higher levels of HCMV products have decreased survival compared to those with lower levels of HCMV expression, supporting the role HCMV has in the pathogenesis and malignant phenotype of GBM (13). Although a better understanding of the mechanisms involved in HCMV onco-modulation is needed, HCMV antigens can be targeted by host-derived T cells using adoptive cellular transfer and represent an important target in immunotherapy for GBM.

## Expanding Adoptive Cellular Therapy Using CMV-Specificity as a Platform

Targeting HCMV antigens displayed on MHC molecules through the native, CMV-specific T-cell receptor (TCR) is a powerful avenue for targeting HCMV-positive GBM using CTLs. Targeting CMV-positive GBM using polyclonal CMV-specific CTLs affords a way to enhance survival, expansion, and cytotoxicity of tumor-directed CTLs because of stimulatory signaling that occurs with binding the native TCR. However, tumors frequently escape immune targeting by downregulating MHC machinery or co-stimulatory signaling molecules required for T-cell mediated killing. Furthermore, targeting a single TAA is generally not clinically effective because of numerous escape mechanisms and the immunosuppressive tumor microenvironment, described below.

Using polyclonal CMV-specific CTLs as a platform, additional targeting through adding chimeric antigen receptors (CAR) specific for a separate TAA is possible. CAR are artificial cell-surface receptors composed of an epitope-binding extracellular domain and an intracellular signaling domain. The extracellular domain contains a single-chain variable fragment (scFv) incorporating light and heavy variable chains of a monoclonal antibody joined by a flexible linker. The scFV can be engineered to target larger peptides, carbohydrates, or glycolipids in an HLA-independent manner, unlike the TCR, which is restricted to smaller peptides that have undergone cytoplasmic processing and display on MHC machinery.

For optimal activation, all T cells require signaling through binding both their TCRs as well as co-stimulatory molecules, primarily CD28. Initial CAR T cells incorporating only the CD3-ζ signaling remained dependent on binding co-stimulatory molecules. Because most tumors do not express co-stimulatory factors, the intracellular domain of the CAR can be enhanced by adding co-stimulatory signaling domains, allowing a way of circumventing downregluation of these molecules on tumor cells.

To date, a limited number of patients have received CMV-specific adoptive cellular therapy as treatment for high-grade glioma, summarized in Table 1. One patient with recurrent GBM received adoptive transfer of expanded CMV-specific T cells and temozolomide with no evidence of progression for 17 months (33). Recently, CMV-specific T cells were successfully expanded from 13 of 19 patients with recurrent GBM (68.4%). Eleven patients received combination therapy consisting of T-cell infusion and chemotherapy without significant toxicities related to T-cell treatment. Whereas the historical median survival in patients with recurrent GBM is less than 6 months, the median overall survival in this group was 403 days, and four out of 10 patients that completed the treatment remained progression-free during the study period (32).

**Table 1:** **Patients receiving CMV-specific adoptive cellular therapy for recurrent or progressive GBM**.

Investigator	Intervention	No. of patients	Timeframe	Outcomes
Ahmed et al. (36)	Adoptive transfer of autologous CMV-specific T cells genetically modified with her-2 specific CAR	15	2010-present, not recruiting	Pending
Crough et al. (33)	Adoptive transfer of autologous HCMV-specific T cells and temozolomide	1	Immunotherapy in 2010	Improved response on MRI, stable clinically after 17 months
Schuessler et al. (32)	Adoptive transfer of autologous HCMV-specific T cells	11	2009–2014	Median overall survival 403 days (range 133–2,428). Time to progression after infusion was 108 to >1,783 days (median, 246). Four of 11 patients remained progression-free
Sampson et al. (37)	Adoptive transfer of autologous HCMV-specific T cells ± pp65-LAMP mRNA loaded dendritic cells	12	2008-present, not recruiting	Pending

Building upon the platform of CMV-specific CTLs that have been shown to kill CMV-positive GBM, we are studying the safety and efficacy of autologous CMV-specific CTLs expressing a HER2-specific CAR in patients with relapsed or refractory her2-positive, CMV-positive GBM (36).

## Immunosuppressive Tumor Microenvironment in GBM

Our ability to exploit HCMV gene expression to target GBM is limited by immunosuppressive characteristics of the tumor and the tumor microenvironment. GBMs secrete inhibitory cytokines, down-regulate antigen presentation and co-stimulatory molecule expression, promote regulatory differentiation in macrophage and APS lineages, and induce immune effector cells to undergo apoptosis. Below, we discuss immunosuppressive and tumor defense mechanisms that must be overcome for CMV-directed therapies or any immunotherapy approach, to be effective in GBM or other solid tumors:

### Immunosuppressive role of glioma stem cells

Within GBM tumors, a subpopulation of GSCs plays a prominent role in creating the immune-suppressive tumor microenvironment (38). These cancer-initiating cells are, by definition, multipotent and propagate the malignant characteristics of GBM and have been shown to be preferentially infected by HCMV *in vivo* (5). GSCs have been characterized by neurosphere formation *in vitro* and expression of CD133, although no marker is completely sensitive or specific for this population. Overall, GSCs tend to exhibit immature antigens, including epidermal growth factor receptor and nestin (39).

Glioma stem cells propagate tumor expansion by inhibiting CTL proliferation and activation, triggering T-cell apoptosis, and inducing FoxP3^+^ regulatory T cells (38). These effects are mediated to a large extent by B7-H1 expression on the tumor cells, as well as release of soluble factors including TGF-β and Galectin-3 (29). While expression of INF-γ is correlated with CTL killing activity, INF-γ also induces upregulation of B7-H1 on tumor cells, which binds to the suppressive cognate programed cell death 1 (PD1) receptor on CTLs (1). Wei et al. (29) found the suppressive effects of B7-H1 and Galectin-3 were diminished on altering the differentiation of the GSCs, suggesting the most potent suppressive mechanisms may come from GSCs. Galectin-3 is produced by glioma cell lines but not normal astrocytes or oligodendrocytes (40).

### Diminished antigen presentation in the CNS and antigen escape

The CNS does not appear to have a robust, immune generating population of APCs, and GBM cells are poor APCs (1). In addition to the paucity of potent APCs in the CNS, MHC-class 1 is not generally expressed in normal CNS cells. Interestingly, GBM cells do express MHC-class 1, and anti-GBM effect has been shown through targeting MHC molecules by HLA-mismatched CTLs (41). This approach is necessarily limited by an eventual host immune response against the allogeneic, HLA-mismatched effector cell. Even though GBMs appear to maintain some degree of MHC expression and antigen-expression, the tumor mutes, and subverts immune activation through inhibitory cytokines and decreased expression of necessary co-stimulatory molecules such as CD80 and CD86 (38).

In general, targeting multiple tumor antigens simultaneously is more effective than targeting a single antigen. While targeting a single antigen may provide an initial response, this strategy may ultimately select for antigen-negative tumor cells and lead to tumor progression (42).

### Downregulation of co-stimulatory molecules and inhibitory surface ligands

Glioma stem cells isolated from human tumors express high levels of MHC-class 1, but they express low levels of CD40, CD86, and MHC-class 2 (29). Inadequate T-cell activation through lack of co-stimulatory signals promotes T-cell anergy and can induce apoptosis (43). In addition to hiding co-stimulatory signals needed for effector cell activation, GBM also expresses inhibitory ligands, particularly B7-H1 (44).

Co-culture experiments with GBM cells show decreased production of IL-2 and interferon gamma (IFN-γ) by autologous CD3^+^ cells (45), and these effects are mediated, in part, through binding the inhibitory co-stimulatory molecule B7-H1. Interestingly, B7-H1 is upregulated by IFN-γ(2) and increases the proportion of CD4^+^FoxP3^+^ Tregs in healthy donor PBMCs incubated from supernatant from GBM cells, making CTL secretion of IFN-γ a double-edged sword as part of the GBM-directed immune response (38). Part of this inhibitory process is mediated by direct contact of the effector cell with the B7-H1 molecule expressed on GBM, as addition of a B7-H1 monoclonal antibody mitigates this effect (29).

### Effector cell apoptosis

In addition to increasing the proportion of regulatory CD4^+^ T cells, GBM induces autologous T cells to undergo apoptosis. T cells undergo apoptosis in co-culture experiments with GBM cells, as well as in experiments where activated and non-activated T cells are incubated with supernatant from GBM cells (29). Although GBM has been reported to express the Fas ligand, the mechanisms through which GBM induce immune cell apoptosis are varied and can also involve B7-H1 signaling through both direct cell contact and cytokine signaling. These pro-apoptotic signals involve Galectin-3, which is produced to a greater extent by GSCs than more differentiated GBM cells (29).

### Subversion of macrophages/microglia and dendritic cells toward a regulatory phenotype

Histology from patient GBM samples frequently demonstrates significant populations of infiltrative macrophages. However, most of these infiltrative macrophages express the M2 phenotype, which is characterized by expression of B7-H1 and overall regulatory, immunosuppressive properties. This ability of the tumor microenvironment to both recruit monocytes/macrophages, and then steer their development along the M2 axis is one of the most powerful evasion strategies protecting GBM from an effective immune response (23, 46).

Although microglia may have some role as an antigen-presenting cell in the CNS, dendritic cells are also prevalent in resected GBM tumors. However, most dendritic cells in the CNS are plasmacytoid dendritic cells, which exhibit a regulatory role compared with myeloid dendritic cells (47). Through promoting the M2 phenotype in monocyte differentiation and protecting against the presence of professional APS, the GBM microenvironment can significantly impair activation and cytotoxicity of effector cells (23).

### Immunosuppressive cytokines and signaling

Many of the above effects, including promoting an M2 phenotype among CNS macrophages/microglia, stem from activation of the STAT3 pathway. In GBM, STAT3 activation induces an immunosuppressive microenvironment by suppressing macrophage activation and decreasing expression of MHC-II, CD80, CD86, and IL-12 in dendritic cells (Wei GBM cancer initiating). IL-6 and epidermal growth factor upregulate STAT3 by phosphorylation. Phosphorylated STAT3 (p-STAT3), which is overexpressed in most gliomas translocates into the nucleus and induces a variety of transcriptional factors, including IL-10. In turn, IL-10 adversely influences Th1-mediated cytotoxic immune responses at multiple levels and is essential for regulatory T-cell function.

Overall, this subversion of a Th1-directed response is a common effect of the immunosuppressive mechanisms of GBM and other tumors. Interestingly, IL-21 appears to promote CD4^+^ T cells along Th1 differentiation and may enhance their anti-tumor effects. GBM cells were modified to secrete IL-21 prior to being implanted into mice. These mice rejected subsequent GBM injections, and some mice with established GBM were salvaged by receiving IL-21-expressing tumor cells (48).

## Alternate Approaches to Cellular Therapy Targeting HCMV in Gliomas

The presence of HCMV in gliomas, including in GSCs, provides antigenic targets for cellular therapy, but can also be used as a mechanism for disrupting tumorigenesis by interfering with important HCMV processes and pathways. The impact of valganciclovir in patients with GBM demonstrates how activity against CMV can translate into clinical response against GBM (49).

In a study of 42 patients with GBM, the addition to valganciclovir in a randomized, double-blind, placebo-controlled trial showed significantly prolonged overall survival at 4 years in patients who received at least 6 months of ganciclovir (50). Valganciclovir has also been shown to slow HCMV-positive medulloblastoma growth in murine models (51).

Similarly, cyclooxygenase-2 (COX-2) inhibition has shown efficacy in slowing tumor pathogenesis in GBM. COX-2 upreglation by the HCMV-protein US28 represents one way in which HCMV contributes to GBM pathogenesis. These pathways involve VEGF and STAT-3. COX-2-derived prostaglandins, particularly PGE_2_, upregulate VEGF and STAT-3 production, ultimately promoting tumor angiogenesis. Multiple studies, including large retrospective analyses, suggest that regularly taking conventional NSAIDs or aspirin significantly reduce the relative risk of death from colon cancer and possibly glioma (52–54). In glioma biology, the COX-2 pathway also induces expansion of a suppressor population of myeloid cells in the glioma tumor microenvironment, where they limit CTL infiltration and anti-tumor effect (53). An improved understanding of these pathways and how they can be manipulated to shift the balance in favor of anti-tumor effect could improve treatment options for gliomas.

## Improving Adoptive Cellular Therapy for GBM

A growing number of glioma-associated antigens and our ability to redirect CTLs to target these antigens – either through the native TCR or a CAR – make adoptive cellular therapy a powerful tool against gliomas. The presence of HCMV within a high proportion of GBM creates an appealing platform for CTL-directed killing, which can be expanded upon by targeting additional epitopes through CARs, as described above.

While combinatorial targeting can prevent antigen escape, the immunosuppressive tumor microenvironment limits the efficacy of cellular therapies for HCMV-positive gliomas. To improve the clinical effectiveness of immunotherapy, effector cells must either be resistant to the deleterious effects of the tumor cells and tumor microenvironment, or the signaling patterns and immune cell phenotypes within the tumor microenvironment must be shifted to favor an anti-tumor immune response.

Within the tumor microenvironment, CTLs are exposed to regulatory cytokines and regulatory ligands expressed by the tumor cells and neighboring, tolerant immune cells. A number of molecular modifications to the CTL itself have emerged and can be improved upon. In successive generations of CARs, the intracellular signaling domain has been improved to provide co-stimulatory signaling artificially upon CAR binding of its target ligand, even when the target cell has downregulated traditional co-stimulatory molecules. Double-negative receptors, which bind inhibitory ligands on tumor cells or regulatory immune cells, are artificial receptors that preferentially bind the immunosuppressive signal but decouple this binding from any immunosuppressive effect. Double-negative receptors that bind TGF-β have been developed and help render CTLs impervious to its effects (4, 55). Developing double-negative receptors for PD-L1 and other immunosuppressive members of the B7 family, or downregulating natively expressed receptors for these and similar molecules on the effector cell, could improve longevity or cytotoxicity of CTLs targeting HCMV on GBM.

Tumorigenesis and disease progression requires the subversion, and often recruitment, of the host immune system by the tumor. The tumor’s ability to control immune checkpoints and shift endogenous immune cells toward a regulatory phenotype are central to its ability to persist and progress. Recent efforts targeting the PD1/PD-L1 axis using monoclonal antibodies demonstrate that anti-tumor effect is possible by shielding endogenous immune cells from immunosuppressive signaling (56, 57). Perhaps the greatest promise for immunotherapy lies with interventions that can reactivate or reprogram an endogenous host immune response against the tumor. Significant work investigating the M1/M2 polarization axis and similar pathways involved in dendritic cell, CD4^+^ T-cell and CD8^+^ T cells programing in the tumor microenvironment is ongoing. Perhaps by manipulating these tumor-corrupted pathways that immobilize and then subvert the host immune response, immunotherapy can cure patients with glioma and other terrible diseases – not necessarily through effector cells manipulated *ex vivo* or exogenous cells, but by reactivating a host army of endogenous immune cells against the tumor.

At this time, multimodal approaches involving not only chemotherapy, surgery, or radiation, but also various forms of immunotherapy, offer the greatest promise for improved clinical efficacy against GBM and other solid tumors. The presence of HCMV and the inherent ability of CTLs to target virus-derived epitopes on glioma cells offer a platform to improve current immunotherapy for GBM. With an improved ability to create and sustain an anti-tumor effect within the tumor microenvironment and potent TAAs such as HCMV, we are optimistic that immunotherapy for GBM and other tumors will cure patients of these terrible diseases.

## Conflict of Interest Statement

The authors declare that the research was conducted in the absence of any commercial or financial relationships that could be construed as a potential conflict of interest.
